# Thoughts on the Effects of Age on the Human Constitution

**Published:** 1847-02

**Authors:** 


### BIBLIOGRAPHICAL NOTICES.

---

ART. VI.—*Thoughts on the Effects of Age on the Human Constitution. A Special Introductory.* By CHARLES CALDWELL, M.D., &c.

*Lecture on Obstetrics and the Diseases of Women and Children.* By GUNNING S. BEDFORD, M.D,, Professor of Obstetrics and the Diseases of Women and Children in the University of New York.

The elaborate lecture of Professor Caldwell opens with the following remarks:

"The vital condition is not only *divisible;* it is actually *divided, by nature* herself into *two forms*—a *latent* or *dormant*, and an *overt* or *active* one. Of these, the former shows itself in vegetables, especially in their *seeds*, and in multitudes of species of the lower orders of animals, when in a state of hybernation. It is a well known fact, that many of these forms of living matter have continued in a condition of *dormant vitality* for hundreds and thousands of years. And to prove that they positively *were* in that condition, they have been subsequently awakened to *active* vitality by suitable stimulation. There are now *peas* and *wheat*, in certain parts of Europe, the products of seeds discovered in Herculaneum, where they had lain dormant for nearly *two thousand*, and in the catacombs of Egypt, where they had lain in a like condition, upwards of *three thousand* years. And, had those seeds not been in a *vital* condition, during this protracted perion, I need, hardly add, that they could not have been roused into *overt* life, by *any* kind, or *all* kinds of stimulation that could have been employed for the purpose. Even an *attempt* to stimulate *dead matter* would involve an absurdity. Nor, had they not been held together by the *vital principle*, would they have retained their form a twentieth part of the time that they *slept in their dormitories*."

Man knows nothing of this dormant condition; active vitality belongs to him, and from its commencement to its close his life "exhibits an unbroken chain of ceaseless action and ceaseless change." But this change occurs only in his material frame, his mind remaining unchangeably the same through every period of his life, from infancy to old age. And yet the mind seems to change. It is feeble in the newborn child, strong in manhood, and again sinks into imbecility with advancing years. "The reason of these changes in mental manifestations does not consist in any alteration in the mind or spirit itself; but in an alteration in the body, the instrument on and by which alone the mind operates *in all* sublunary concerns."

The life of man consists of a number of periods, the characteristics of which are severally pointed out in the lecture. We quote the remarks of the author respecting the first of these periods:—

"At the commencement of their *uterine* period the elements of the future man can hardly be said to possess real solids or real organization. The *germinal vesicle* and the *embryonic cell*, together with all that immediately surrounds them, are nothing but specks of mucus or gelatin, without any shape that can be called *significant*—significant I mean of the figure and character of the being they are to produce. But they are *living* gelatin, and possess a plastic power which it would be hardly extravagant to denominate *marvellous*. It is certainly one of the most singular and striking that is in any case exercised by living matter. It is to this effect.

"The elements of the embryos of all kinds of animals are precisely alike. At least they resemble each other so accurately, that the ablest minute anatomist and observer, armed with the best microscope ever constructed, cannot distinguish one of them from another. The germinal elements of the fish, the reptile, the bird, the quadruped,—and even of man, appear to be *identical* in form and composition, and nearly so in size. Yet do they, in the progress of their development, put on each its generic and specific form, and exhibit its corresponding instinct and qualities. The fish swims, and lives and acts conformably—the reptile crawls — the bird flies—the quadruped walks, runs, and leaps—and man stands erect, with his "*os sublime*," and manifests the superior attributes of his superior organization. Of the vegetables the same is true. Their germinal elements appear to be *identical*. Yet do they put forth in time products marked by that infinite diversity of shape, size, color, taste, and smell which characterizes the countless hosts of the vegetable kingdom."

Of all the periods, decadence is the only one not fixed by nature. Happily, this is left somewhat to our control.—"Each man that enters it," says the lecturer, "is so far master of it that he can either protract or curtail it to an important extent." The author has allusion rather to the mental than the corporeal powers; and yet by temperance, he insists, and insists correctly, the physical decay of man may be averted much longer than it usually is.

Professor Bedford's lecture, although introductory, discusses practical questions of the gravest import, of which the following quotations afford fair specimens:

"Another common error in practice is to impute the various ailments of the female to *falling of the womb;* and hence, if we are to credit what is said on this subject by certain practitioners, *falling of the womb* is the true cause of all female complaints. It would, indeed, be much nearer the truth to affirm that this affection is very commonly the result of carelessness on the part of the physician in not detecting at an early period, a disease of the cervix of this organ, which, by being suffered to progress, terminates in chronic engorgement, thus causing the uterus to descend, and producing a veritable prolapsion. It has been shown by Lisfranc, to whom the profession is under deep obligations for the light he has thrown on the causes and treatment of affections of the womb, that *prolapsus uteri* is rarely a primary disease, but usually consequent upon enlargement of the *cervix*, which, by its increased weight, pulls the uterus into the vagina. With this simple explanation, which undoubtedly is based on facts, it must be evident that the very means usually resorted to for the cure of falling of the womb, are the direct agents of mischief—they not only do no good, but are positively injurious. Every medical man, who possesses a proper knowledge of his profession, is well aware that this disease is extremely rare among unmarried ladies. What, therefore, must be the character of a practitioner, who can see no other cause for the sufferings and ill-health of a young lady, than *falling of the womb*, and consequently subjects her to the most cruel and unjustifiable examination. I have heard of several young creatures in one family, averaging from sixteen to eighteen years of age, condemned to this most shameful desecration of their persons.

"During the last winter, I was requested to visit professionally a married lady from the State of New Jersey. The history of her case was simple and to the following effect:—In June, 1842, she experienced uneasiness in the region of the womb, and slight pain in passing water. There was more or less discharge of mucus from the vagina, and sexual intercourse occasioned at times great distress. These were the incipient and only symptoms of her malady. A physician was consulted, and immediately pronounced the disease *to be falling of the womb*. Pessaries were introduced—abdominal supporters applied, but without affording any relief to the suffering patient; whilst, on the contrary, the pessaries tended to aggravate the pain by the pressure they exerted on the seat of disease. Another practitioner was consulted, and reiterated the opinion already advanced.—Having continued under his care for more than ten months, without deriving the slightest benefit, and experiencing a positive increase in her sufferings—the pain and difficulty in passing water becoming more aggravated—she resolved to visit the city of New York in search of professional advice. She arrived here in December last, and I was requested to see her. On hearing the history of the case, I frankly told her that I did not believe she had *falling of the womb*, for the simple reason that her symptoms were not characteristic of any such ailment. I proposed an examination which was cheerfully consented to, as the lady was most solicitous to obtain relief. The uterus I found in a perfectly healthy condition and in its normal position. In passing my finger along the *urethra*, the patient experienced a sensation of pain, and this circumstance, together with the difficulty of which she complained in passing water, attracted my attention especially to this point. I could detect no disease in the uterus or vagina; in attempting to introduce an ordinary female catheter into the urethra I was completely foiled; and, on minutely examining the condition of this passage, I discovered that the lady's sufferings were entirely due to a *stricture*

*of the urethra.* *Stricture of the female urethra* I had never seen previously to this occasion; and, as far as my knowledge extends, no case of the kind had ever occurred in this country; at least, no record has been made of it. Velpeau, in his great work, cites but three cases of *stricture of the female urethra*, and remarks that its occurrence is extremely rare. In the course of three months, I succeeded in removing the stricture, and the lady returned to her home entirely restored to health.

"On the 10th day of last July a lady, residing in this city, sent by note a request that I should pay her a professional visit. She informed me that, for the last few months, she had been under the care of a practitioner who pronounced her to be laboring under falling of the womb—that he had on one occasion introduced an instrument, (a speculum, I imagine,) which gave her so much uneasiness that, in twelve hours afterwards, a miscarriage was the consequence. I was also informed by the lady that she had been under the necessity of calling repeatedly at the house of this practitioner, his engagements being so numerous that he could not find time to visit her. She had faithfully observed all his directions, but received no benefit from his treatment. In my first interview with this patient, after hearing the above statement, I requested her to give me a full account of her symptoms. She remarked that since the birth of her last child, about twelve months since, she experienced great irritation about her bladder—her nights were much disturbed by more or less desire to pass water—being obliged to leave her bed more than a dozen times during the night. The irritation of the bladder had entirely disfranchised her from all social intercourse—and she could not with any degree of comfort leave her house. This state of things had continued without any palliation to the time of my visit, and her sufferings had produced an evident inroad on a constitution otherwise vigorous. I then asked her if her last labor had been unusually protracted, or whether she had experienced at the time any difficulty about the bladder. To which she replied that her labor was natural and of short duration—but the doctor, who attended her in confinement, in attempting to introduce a catheter into the bladder for the purpose of drawing off the water, gave her much pain, and caused a slight discharge of blood, unaccompanied, however, by any flow of urine. This was all the information I obtained from the patient, and I think you will agree with me that there was nothing in the case to justify the suspicion that her difficulty was attributable to *falling of the womb.* On examination, I found the womb entirely free from disease, and in its natural position. I detected just within the *meatus urinarius* a small ulcer, which was most probably the result of injury inflicted by the catheter. This ulcer I regarded as the only cause of her suffering, and informed the lady with great confidence that in a few days she should be restored to health. On the following day, assisted by Dr. Trantham, of South Carolina, in whose presence the above statement was repeated by the patient, I touched the ulcer with the nitras Argenti—ordered mucilaginous drinks—and, on the fifth day after the operation, she took a long walk without inconvenience; to the present period she has never experienced the slightest irritation or pain about the bladder. Her health has continued excellent, and she is at this time in the sixth month of gestation."

The printers have taken unpardonable liberties with Dr. Bedford's manuscript, as they sometimes do with ours, inserting a letter or changing one, it would seem, simply to please their queer fancies. These are among the small vexations of editors and authors.

